# Early calorie-dense immune nutrition in haemodynamically compromised cardiac patients

**DOI:** 10.1186/cc14479

**Published:** 2015-03-16

**Authors:** S Efremov, V Lomivorotov

**Affiliations:** 1 Research Institute of Circulation Pathology, Novosibirsk, Russia

## Introduction

The aims of present study were to test the hypothesis that early enteral nutrition (EN) with calorie-dense food supplemented with glutamine improves recovery of nutritional status in critically ill cardiac patients and to evaluate their resting energy expenditure (REE).

## Methods

A prospective randomised study of 40 adult cardiac patients undergoing elective cardiopulmonary bypass surgery no more than 24 hours before eligibility assessment, complicated with acute heart failure syndrome. Patients were randomised to receive either standard isocaloric isonitrogenic early EN (standard group, *n *= 20) or immunomodulating early EN (immune group, *n *= 20). The daily energy target was set using REE measured by indirect calorimetry (CCM Express; Medgraphics, St. Paul, MN, USA). Serum prealbumin, transferrin, C-reactive protein, blood lactate and clinical characteristics were analysed.

## Results

**Figure 1:**
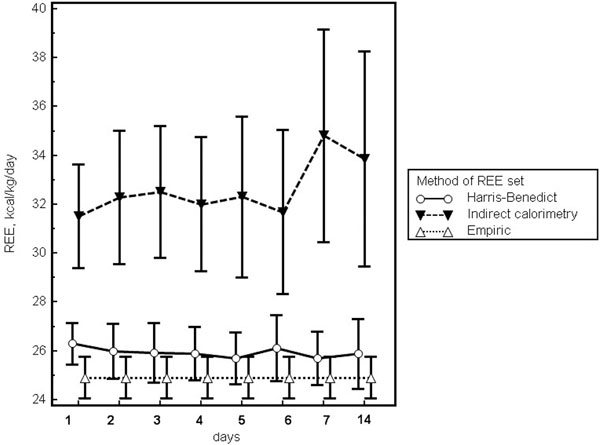
**Indirect calorimetry measured significantly higher resting energy expenditures**.

The actual REE was an average of 6.8 and 7.5 kcal/kg/day higher than the REE calculated using the Harris-Benedict equation and empiric approach (25 kcal/kg/day), respectively (Figure 1). Early EN with immune formula was associated with higher levels of prealbumin concentration on the 14th day (0.13 ± 0.01 g/l and 0.21 ± 0.1 g/l; *P *= 0.04) and transferrin on the 3rd, 5th, 7th, and 14th day (*P *< 0.05) after surgery.

## Conclusion

Haemodynamically compromised cardiac patients have increased REE, which in the absence of indirect calorimetry should be set at 30 kcal/kg/day. Early EN using a calorie-dense immune formula leads to better recovery of nutritional status as assessed by serum protein levels.

